# Deep learning-based lung sound analysis for intelligent stethoscope

**DOI:** 10.1186/s40779-023-00479-3

**Published:** 2023-09-26

**Authors:** Dong-Min Huang, Jia Huang, Kun Qiao, Nan-Shan Zhong, Hong-Zhou Lu, Wen-Jin Wang

**Affiliations:** 1 Department of Biomedical Engineering, Southern University of Science and Technology, Shenzhen, 518055 Guangdong China; 2 The Third People’s Hospital of Shenzhen, Shenzhen, 518112 Guangdong China; 3 Guangzhou Institute of Respiratory Health, China State Key Laboratory of Respiratory Disease, National Clinical Research Center for Respiratory Disease, The First Affiliated Hospital of Guangzhou Medical University, Guangzhou, 510120 China

**Keywords:** Deep learning, Lung sound analysis, Respiratory sounds

## Abstract

Auscultation is crucial for the diagnosis of respiratory system diseases. However, traditional stethoscopes have inherent limitations, such as inter-listener variability and subjectivity, and they cannot record respiratory sounds for offline/retrospective diagnosis or remote prescriptions in telemedicine. The emergence of digital stethoscopes has overcome these limitations by allowing physicians to store and share respiratory sounds for consultation and education. On this basis, machine learning, particularly deep learning, enables the fully-automatic analysis of lung sounds that may pave the way for intelligent stethoscopes. This review thus aims to provide a comprehensive overview of deep learning algorithms used for lung sound analysis to emphasize the significance of artificial intelligence (AI) in this field. We focus on each component of deep learning-based lung sound analysis systems, including the task categories, public datasets, denoising methods, and, most importantly, existing deep learning methods, i.e., the state-of-the-art approaches to convert lung sounds into two-dimensional (2D) spectrograms and use convolutional neural networks for the end-to-end recognition of respiratory diseases or abnormal lung sounds. Additionally, this review highlights current challenges in this field, including the variety of devices, noise sensitivity, and poor interpretability of deep models. To address the poor reproducibility and variety of deep learning in this field, this review also provides a scalable and flexible open-source framework that aims to standardize the algorithmic workflow and provide a solid basis for replication and future extension: https://github.com/contactless-healthcare/Deep-Learning-for-Lung-Sound-Analysis.

## Background

Lung disease has been a leading cause of mortality worldwide for many years, especially since the onset of corona virus disease 2019 (COVID-19) [1–3]. Various clinical methods have been developed to diagnose and evaluate lung health conditions, including computed tomographic scans, chest X-rays, and pulmonary function tests (PFTs) [4, 5]. However, these methods are often limited to high-end clinics due to their complexity and high costs [6]. In contrast, auscultation offers a non-invasive, low-cost, and portable way of working where paramedics use a conventional acoustic stethoscope to diagnose lung diseases, including asthma, chronic obstructive pulmonary disease (COPD), and pneumonia [7–9], based on the patient's lung sound.

Although the stethoscope has been widely used in clinics, it has several associated challenges. First, the interpretation of lung sounds requires a trained paramedic, limiting stethoscope use in low-resource areas [10]. Second, the medical-decisions made based on auscultation are subject to inter-listener variability in proficiency [11]. The subjectivity of the diagnosis is further amplified by the lack of a recording function in the conventional stethoscope that prevents other personnel from analyzing the sounds heard during the consultation [12]. These challenges need to be resolved to improve the quality and efficiency of lung disease diagnosis.

To this end, the digital stethoscope has been developed to record lung sounds by digitizing acoustic signals [13]. It enables the visualization and retrospective analysis of lung sounds. In addition, wireless transmission (e.g., Bluetooth or WiFi) allows it to be used for remote diagnosis, further increasing the convenience of application [14–16]. The emergence of digital stethoscopes combined with related physics study [17] has contributed to our understanding of lung sounds including, their production, transmission, and characteristics under healthy and pathological conditions [18].

Based on this understanding, the recognition of lung sound patterns using machine learning has been achieved, providing an objective and quantitative method for lung health assessment [19]. Earlier studies focused on the feature engineering of lung sounds and exploitation of shallow machine learning tools for abnormal lung sound detection [20]. Zhang et al. [21] conducted a clinical trial showing that support vector machine (SVM)-based diagnosis performed better than general pediatricians in abnormal lung sound detection, achieving an accuracy of 77.7% and 59.9% for crackles and wheezes, respectively. This demonstrates the potential of machine learning in intelligent lung sound recognition.

More recently, deep learning-based models were proposed to detect the patterns related to lung diseases and distinguish abnormal lung sounds from normal ones and have shown promising performance [22]. Compared with shallow machine learning, most deep learning-based methods adopt an end-to-end learning approach to automatically learn the representation of lung sounds from raw acoustic signals without the need for handcrafted feature engineering. They can also leverage transfer learning to increase the adaptability of the learned models in new environments, which reduces the amount of data needed for training [23, 24]. It is important for clinical applications due to the difficulty of acquiring a large amount of patient data. Pham et al. [25] applied convolutional neural networks (CNNs) to learn temporal-frequency information from spectrograms, and achieved 89% specificity and 82% sensitivity in normal and abnormal lung sound classification. Perna et al. [26] used recurrent neural networks (RNNs) to mine the context information of lung sounds over time, obtaining an accuracy of 99% in recognizing COPD patients. In addition, Altan et al. [27] proposed a deep belief network-based model combined with a three-dimensional (3D)-second order difference plot of lung sound signals to distinguish the severity of COPD patients. These methods demonstrate the feasibility of implementing deep learning-based intelligent stethoscopes that can automate the detection of pulmonary disease and its severity. Moreover, deep learning-based quantitative results overcome the disadvantages of subjective auscultation diagnosis caused by inter-listener difference and the need for clinical proficiency, thus supporting medical diagnosis and treatment. Thus, deep learning-based approaches can significantly improve the quality of healthcare in underdeveloped countries with limited clinical resources; examples of their applications include community-acquired pneumonia detection and the domiciliary management of COPD.

**Fig. 1:**
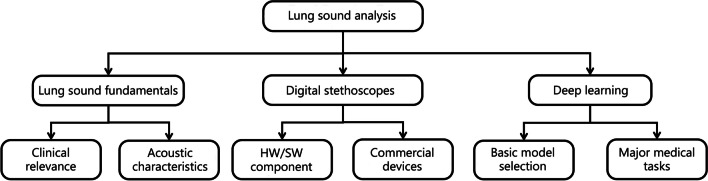
An overview of deep learning in lung sound analysis. The fundamentals of lung sounds include clinically relevant knowledge and its acoustic characteristic, which guides and motivates the design of the digital stethoscope in hardware and software. In turn, the application of digital stethoscope-based deep learning methods verifies the understanding of observations

To increase the understanding of deep learning-based lung sound analysis, in this paper, we systematically review deep learning methods proposed for lung sound analysis. This review, organized as shown in Fig. 1, outlines the system of lung sound analysis, including the pathological fundamentals of lung sounds, existing digital stethoscopes, and deep learning-based methods. The fundamentals of lung sounds guide and motivate the design of reasonable deep learning methods, and in turn, the application of digital stethoscope-based deep learning methods verifies the understanding of observations. In contrast to previous reviews [6, 19, 28–31], this paper emphasizes the applications of deep learning-based lung sound analysis, including the system framework, basic model selection, and the advancement of deep methods in respiratory medical tasks, also highlighting the challenges that need to be overcome. The main contributions of this review are as follows: (1) It provides an in-depth review of the fundamentals of lung sounds under normal and pathological conditions that motivates the design of deep-learning models and guides the design of signal processing algorithms (spectrograms, typical signatures, and their definitions); (2) It provides a thorough overview of the algorithmic framework of deep learning-based lung sound analysis, with a detailed introduction to each processing step, including the pros and cons of deep models and challenges they face; and (3) It provides a unified open-source deep learning-based framework that aims to standardize algorithmic components and establish a strong base that facilitates replication, benchmarking, and future extension.

The remainder of this paper is structured as follows. First, the fundamentals of lung sounds are presented. Then, the existing digital and wireless stethoscopes that can be used for clinical purposes are described, followed by an overview of the framework of deep learning in lung sound analysis including the main tasks, preprocessing, public datasets, and related research. Furthermore, an open-source framework for deep learning-based lung sound analysis is introduced. Finally, the conclusions of this review are presented.

## Fundamentals of lung sounds

This section provides an overview of lung sound to improve our understanding of its definitions, as summarized in Table 1, which is important for designing and implementing methods for lung sound analysis.

**Table 1:** The understanding of normal and abnormal lung sounds

Categories of lung sounds	Produce/Cause	Timing	Acoustics characteristics	Associated disease
Normal				
Tracheal	Turbulent airflow through pharynx and glottis	Both inspiration and expiration	Hollow, non-musical, harsh; High-pitch, 100–5000 Hz, drop at 800 Hz	–
Bronchial	Airflow traversing from trachea to the main airways	Inspiration, mostly expiration	Soft, non-musical, tubular; High-pitch, similar to tracheal	–
Vesicular	Airflow through smaller airways and alveoli	Inspiration, early expiration	Soft, non-musical; Low-pitch, 100–1000 Hz, drop at 200 Hz	–
Bronchovesicular	Airflow through bronchi and alveoli	Both inspiration and expiration	Frequency between vesicular and bronchial	–
Abnormal				
Fine crackle	Explosive opening of small airways or the alveoli	Mid-to-late inspiration, occasionally expiration	Explosive, non-musical; High-pitch, 650 Hz; Duration: 5 ms	Interstitial lung fibrosis, pneumonia, pulmonary fibrosis, asbestosis
Coarse crackle	Air bubble in larger airways	Expiratory, mostly early inspiratory	Explosive, non-musical; Low-pitch, 350 Hz; Duration: 15 ms	COPD, bronchiectasis, asthma
Pleural rub	Pleural membrane rubbing against each other	Biphasic	Non-musical, rhythmic; Low-pitch, 350 Hz; Duration: 15 ms	Pleural inflammation, pleural tumors
Wheeze	Airflow limitation, airway narrowing	Inspiratory, mostly expiration	Musical, sibilant; High-pitch, > 100 Hz; Duration: > 80 ms	COPD, asthma, foreign body
Rhonchi	Thickening of secretions in bronchial tree	Inspiratory, mostly expiratory	Musical, sibilant; Low-pitch, 200 Hz; Duration: > 80 ms	Bronchitis, COPD
Stridor	Upper airway obstruction	Mostly inspiratory, sometimes both	Musical, sibilant; High-pitch, 500 Hz; Duration: > 250 ms	Epiglottitis, foreign body, croup, laryngeal oedema

“-” none, *COPD* chronic obstructive pulmonary disease

**Fig. 2:**
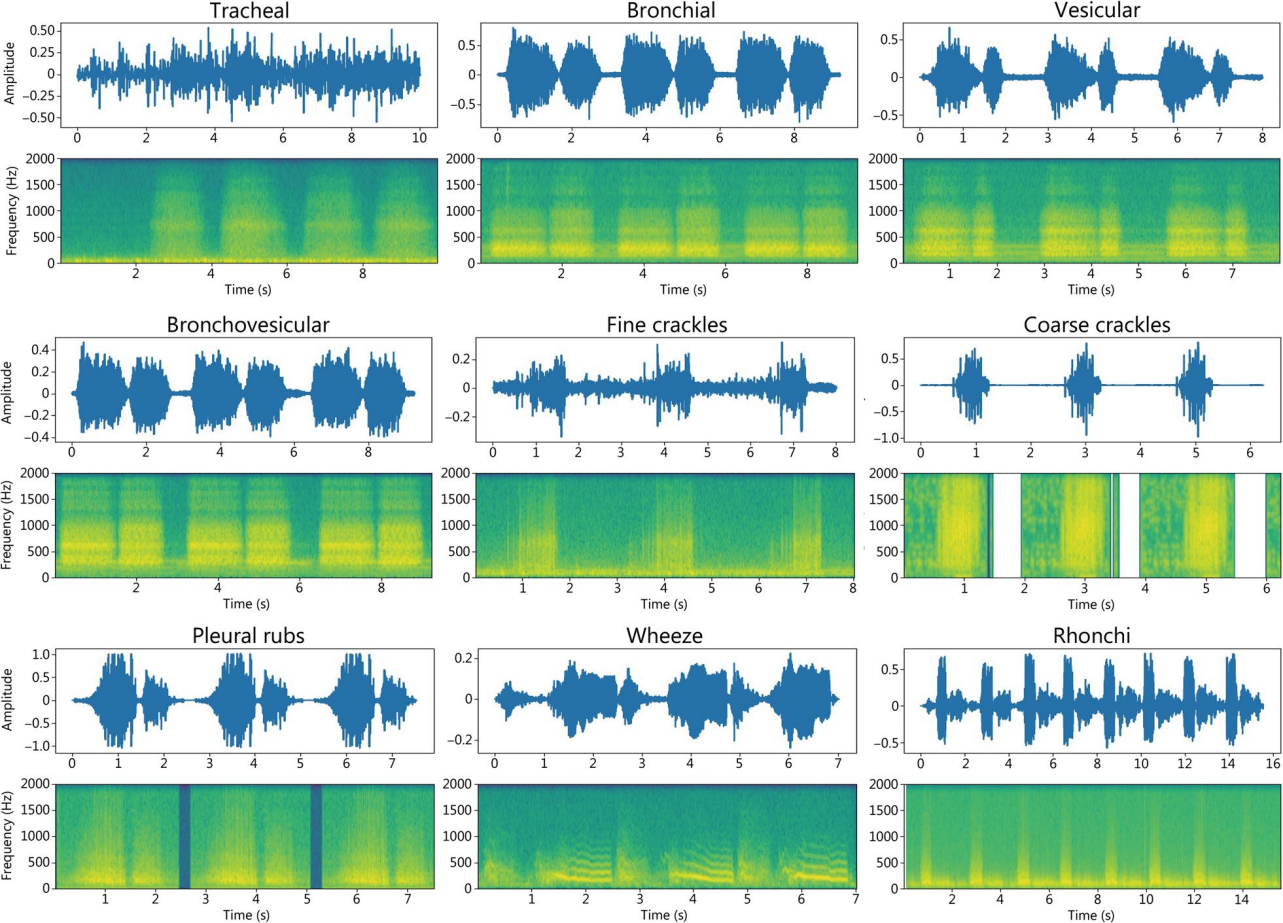
Lung sound demo. In each example, the upper panel shows the acoustic signal and the lower panel shows the corresponding spectrogram

Lung sound, also termed respiratory sound, can be categorized into two types according to the health condition: (1) normal lung sound, which refers to the sounds generated by the airflow passing through the healthy respiratory system [32]; (2) abnormal lung sound, which is generally caused by lung diseases, exemplified by the presence of additional sounds overlaying the normal lung sound, the absence or reduction of normal lung sound, and asymmetry between left and right lung sounds [28]. Figure 2 portrays these separately.

### Normal lung sound

Normal lung sound mostly consists of tracheal, bronchial, vesicular, and bronchovesicular sounds [33]. The differences between regarding the mechanism of generation, auscultation location, appearance timing, and acoustic characteristics are shown in Table 1.

Tracheal sound is produced by the turbulent airflow passing the tracheal tissues of the respiratory system [34]. When auscultation is carried out over the trachea, particularly above the sternum, this sound can be heard clearly during both the inspiratory and expiratory phases. The tracheal sound lasts for a similar duration in both phases, and the pause between the two phases is obvious [35]. Since its transport occurs in the straighter part of the trachea with a larger diameter, the tracheal sound is typically high-pitched, hollow, non-musical, harsh, and louder than other normal lung sounds [36, 37]. The normal tracheal sound has a wide energy distribution of 100–5000 Hz, and the energy usually drops at 800 Hz [38].

Bronchial sound is generated by the airflow traversing from the trachea to the main airways, and can usually be heard near the second and third intercostal spaces [37]. Like the tracheal sound, it appears in both phases but mainly in the expiratory phase, twice as long as in the inspiratory phase [39]. In general, the bronchial sound is generally soft, non-musical, loud, high-pitched, and tubular, with a similar frequency energy distribution as the tracheal sound [28, 40].

Vesicular sound is created by the airflow passing through the smaller airways and alveoli (tiny air sacs) in the lungs [41]. It is audible in most of the lung fields across the whole inspiration phase and the early expiration phase [35, 42, 43]. The vesicular sound is typically soft, non-musical, and low-pitched and its frequency range is from below 100–1000 Hz with an energy drop at 200 Hz [40, 44].

Bronchovesicular sound can be heard between the scapulae in the posterior chest, and in the central region of the anterior chest [40]. It has a similar duration in the expiratory and inspiratory phases [39]. In sound analysis, the bronchovesicular sound is softer than the bronchial sound but approximates the tubular sound, similar to the sound between the bronchial and vesicular sounds. Additionally, the frequency band of bronchovesicular sounds is between that of vesicular and bronchial sounds [44].

### Abnormal lung sound

Abnormal lung sounds can be distinguished as discontinuous and continuous abnormal sounds according to their acoustic properties. The former has a shorter duration of less than 25 ms including fine crackle, coarse crackle, and pleural rub, whereas the latter typically has a longer duration of more than 250 ms [28], including wheeze, rhonchi, and stridor. Table 1 presents a description of these lung sounds in terms of their causes, appearance timing, clinical characteristics, acoustic characteristics, and the associated diseases.

Fine crackle arises due to the explosive opening of small airways or alveoli that were previously collapsed or closed [45]. It is commonly audible in mid-to-late inspiration and sometimes in the expiration phase, changing or disappearing with the body position [35]. Clinical study has reported that fine crackle is caused by several diseases, such as interstitial lung fibrosis and pneumonia [35]. It can be used as a biomarker for detecting specific diseases such as idiopathic pulmonary fibrosis and asbestosis, showing good sensitivity and specificity [46]. Fine crackle presents as high-pitched (close to 650 Hz), non-musical, and explosive, with a duration of nearly 5 ms [47].

Coarse crackle is probably caused by air bubbles in larger airways that open and close intermittently [48]. Upon auscultation, it can be heard in both phases, mostly in the early inspiratory phase [49]. Due to intermittent airway opening, it is associated with some obstructive diseases, for example, COPD, bronchiectasis, and asthma [28, 50]. In contrast to fine crackle, coarse crackle is low-pitched (close to 350 Hz) and has an approximative duration of 15 ms [51].

Pleural rub is generated by the rubbing of the pleural membranes against each other and is relevant to pleural inflammation and pleural tumors [35]. It is typically biphasic with the expiratory sequence of sounds mirroring the inspiratory sequence [37]. Pleural rub is non-musical, rhythmic, and low-pitched (< 350 Hz). Its duration is longer than 15 ms.

Wheeze is produced by airflow limitations due to airway narrowing and is normally detected in both phases, mostly in the expiration phase [52]. Wheezing sounds are typically caused in asthma and COPD, possibly by a foreign body (e.g., a tumor) blocking the airway [35]. In general, wheeze is musical, sibilant, and high-pitched (more than 100 Hz). Its duration is generally more than 80 ms [53].

Rhonchi are related to the thickening of secretions in the bronchial tree and can be heard mostly in the expiration phase and sometimes in the inspiratory phase. Rhonchi are reported to be associated with bronchitis and COPD [35]. The acoustic characteristics of rhonchi are similar to those of wheeze sounds but with a relatively low pitch (< 200 Hz) [53].

Stridor is created by the turbulent airflow in the bronchial tree, which is relevant to upper airway obstruction. Upon auscultation, it can be detected mostly in the inspiration phase, but in certain situations, it can be heard in both phases [28]. Diseases related to upper airway obstruction may cause stridor, including croup and laryngeal edema. Stridor is a sibilant and musical sound that has a high pitch above 500 Hz with a duration longer than 250 ms.

## Digital stethoscopes

For deep learning-based lung sound analysis, the data acquisition process depends on digital stethoscopes that record the lung sound by converting acoustic waves into electrical signals. Thus, this section focuses on digital stethoscopes currently available in the market and widely used in clinics, with an emphasis on their limitations and potential directions for improvement.

### Implementation of digital stethoscopes

**Fig. 3:**
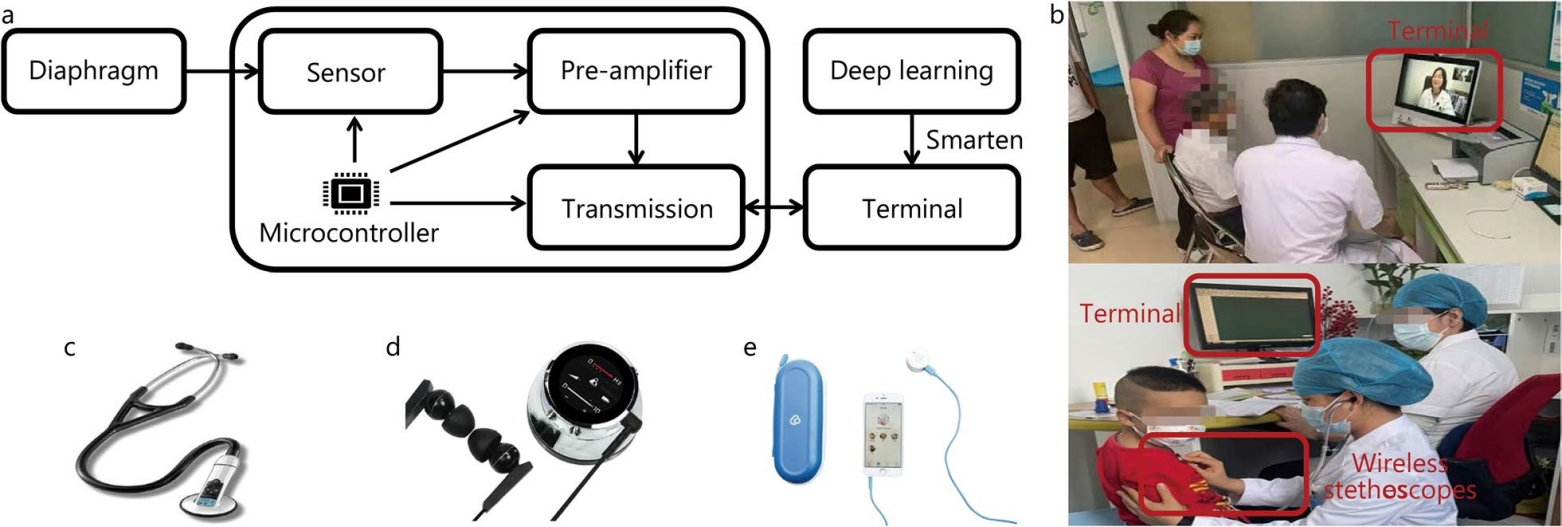
Digital stethoscopes. **a** Implementation of wireless stethoscopes; **b** Telemedicine; **c** 3 M LITTMAN 3200; **d** Thinklabs; **e** Clinicloud

A digital stethoscope generally consists of a diaphragm, sensor, pre-amplifier, microcontroller, and transmission module [54, 55], as shown in Fig. 3. Its workflow is as follows in Fig. 3a, b: first, the diaphragm is placed on the chest piece to capture the sound wave of the internal body [56]. Then, either piezoelectric sensors or electret microphones are commonly used to convert the sound waves into electrical signals [57, 58]. The pre-amplifier enhances the extremely weak acoustic signal that is picked up by the sensor [59]. Next, the microcontroller processes the amplified signal, which includes controlling the audio processing circuitry and managing the user interface and display. Finally, under the control of the microcontroller, the transmission module (e.g., Bluetooth), transmits data to the terminals in a lossless way as far as possible [60, 61].

### Available digital stethoscopes

Here, we focus on digital stethoscopes that have been used as clinical devices, including 3 M LITTMAN 3200, Thinklabs digital stethoscope, and Clinicloud digital stethoscope, as shown in Fig. 3c–e.

#### 3M LITTMAN 3200

The most popular stethoscope, it amplifies 24 times for acoustic signals with a denoised module and offers a mobile applications system for lung health management. A clinical trial showed that the diagnostic accuracy of medical interns was improved upon using LITTMAN 3200 compared to the traditional acoustic stethoscope [62]. Some studies also used machine learning to automatically detect abnormal lung sounds and diagnose lung diseases in offline clinical studies, wherein the 3M LITTMAN 3200 was applied to collect and transmit lung sounds [10, 63, 64].

#### Thinklabs digital stethoscope

This is a tube-free device that can amplify acoustic signals 100-fold, remove noises that have different frequency bands by using multiple frequency filters, and provide a mobile APP. This stethoscope has been clinically investigated for pneumonia detection [65] and the analysis of the frequency characteristics of normal lung sounds [66].

#### Clinicloud digital stethoscope

This stethoscope has been designed without the function of signal amplification. It was used in a clinical trial at Melbourne Hospital and showed accurate abnormal sound detection (ASD) in children [67].

### Limitations and future improvements

Although the abovementioned stethoscopes are capable of recording and transmitting lung sounds, they still face some challenges. First, the high price of existing digital stethoscopes limits their scope of application in low-resource areas. Such areas desperately need low-cost and easy-to-operate medical devices since they cannot afford expensive equipment and manpower. Second, the available commercial digital stethoscopes are single-channel devices, making it difficult to monitor the left and right lungs synchronously. The diagnostic accuracy of single-channel devices can be improved by extending them to multiple channels [68–70]. Third, the difference in sound quality between these stethoscopes may cause deviations in the performance of algorithms for lung sound analysis [71]. Gairola et al. [72] performed device-based fine-tuning to improve the quality of detection; however, it is not practical to tune all these devices.

To solve these challenges, future research should focus on the implementation of low-cost and highly-reliable digital stethoscopes. Specifically, the development of each component of the device can facilitate this goal. For example, the expensive commercial diaphragm can be replaced with 3D-printed materials [73]. For signal transmission, the lung sound signal can be transmitted by matured technologies such as Bluetooth Low Energy [74] and Zigbee [75], allowing stethoscopes to be a part of the Internet of Medical Things to provide more comprehensive lung health assessments [76]. Furthermore, the development of wearable devices is also conducive to all-weather lung health monitoring. Meanwhile, the endurance and intelligence of digital stethoscopes need to be improved by introducing new technologies regarding the battery, processor, and embedded algorithms to cope with medical situations in low-resource areas.

## Deep learning in lung sound analysis

**Fig. 4:**
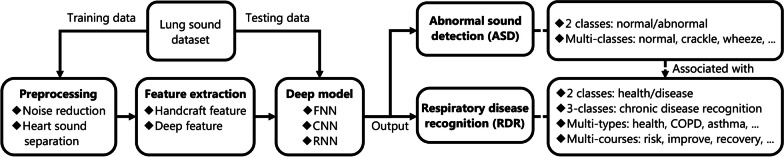
Deep learning-based framework for lung sound analysis. For two different medical tasks (ASD and RDR), the training set is used to construct the model including the steps of preprocessing, feature extraction, model selection. Finally, the test set is used to evaluate the performance of model. FNN fully connected neural network, CNN convolutional neural network, RNN recurrent neural network, COPD chronic obstructive pulmonary disease

This section reviews deep learning studies for lung sound analysis including the system framework, common datasets, preprocessing, feature extraction, and deep learning methods designed for different medical tasks, as shown in Fig. 4.

### System framework

Clinically, auscultation results depend on the doctor's interpretations of lung sounds, which are often subjective based on the proficiency of the listener. As a result, the clinical decisions made for the same patient may vary between physicians, promoting misdiagnosis and missed diagnosis. To solve this issue, machine learning methods (SVM, CNN, and random under-sampling boosting) have been proposed in different clinical contexts to provide quantitative and objective results on different types and degrees of lung disease [21, 77, 78]. However, most shallow machine learning-based lung sound analysis methods were evaluated based on a self-collected dataset of only a few subjects that was saturated at a low accuracy of approximately 80% [79–81].

Recently, deep learning has shown great potential in lung sound analysis, with a more accurate and robust performance compared with shallow machine learning [82]. Its improved performance may be attributed to the following features. (1) Representation: deep learning methods automatically learn task-relevant features in a data-driven manner without the need for manual feature engineering, and the learned features can capture complex patterns and structures in the raw data [22]; (2) Context information: deep learning methods show the advantages of capturing temporal context information, such as RNNs, which is significant for lung sound analysis in mining periodic lung sound changes caused by disease [26]; (3) Transfer learning: deep learning methods can use the common knowledge shared with related fields (e.g., AudioSet [83], a large audio dataset) to improve lung sound analysis, which reduces the amount of data required for training [24]. This property is significant for clinical applications since clinical data are often scarce due to the challenge of organizing clinical trials.

Generally, most deep learning-based lung sound analyses follow the paradigm of sequentially executing data acquisition and preprocessing, feature extraction, and classification. First, a digital stethoscope is used to collect lung sound data, following which preprocessing is applied to suppress environmental noise in the recorded lung sound signals. Thereafter, feature extraction is used to convert high-dimensional preprocessed lung sound data into a lower-dimensional space to obtain a more discriminative representation. Finally, the classifier is designed to create a mapping between the features and classes of relevant diseases.

### Datasets for lung sound analysis

To evaluate performance, many deep learning-based lung sound analysis methods were benchmarked on public datasets for a fair comparison. The public lung sound datasets [84–88] are summarized in Table 2. The most widely used dataset is the ICBHI 2017 Respiratory Sound Database [84] which consists of 920 recordings from 126 subjects who were diagnosed with respiratory pathological conditions, such as pneumonia, bronchiectasis, bronchiolitis, and COPD. Those recordings had different sampling rates (e.g., 4000 Hz, 10,000 Hz, and 44,100 Hz) and their duration ranged from 10 to 90 s. For annotation, the medical teams labeled the beginning and end of the breathing cycles in each recording as well as the presence/absence of crackles and wheezes. This dataset collected 6898 breath cycles, with 3642 normal cycles, 1864 with crackles, 886 with wheezes, and 506 with both, where the cycle duration of all recordings varied from 0.2 to 16 s, with a mean duration of 2.7 s.

**Table 2:** Public lung sound datasets

Dataset	Subject	Audio recording	Duration	Annotation	Diagnosis
ICBHI 2017 [84]	126	920	10–90 s	Normal, crackle, wheeze, both crackle and wheeze	Lower respiratory tract infections, upper respiratory tract infections, pneumonia, COPD, asthma, bronchiolitis, bronchiectasis, cystic fibrosis
Fraiwan et al. [85]	112	112	5–30 s	Inspiratory, expiratory, wheezes, crackles, normal	Normal, asthma, pneumonia, COPD, bronchitis, heart failure, lung fibrosis, pleural effusion
HF_Lung_V1 [86]	261	9765	15 s	Inhalation, exhalation, stridor, rhonchus, crackle	Acute respiratory failure, chronic respiratory failure, acute exacerbation of COPD, COPD, pneumonia, acute respiratory distress syndrome, emphysema
HF_Lung_V2 [87]	303	14,138	15 s	Inhalation, exhalation, stridor, rhonchus, crackle	Acute respiratory failure, chronic respiratory failure, acute exacerbation of COPD, COPD, pneumonia, acute respiratory distress syndrome, emphysema, long-term mechanical ventilation
RespiratoryDatabase@TR [88]	77	77	> 17 s	COPD 0–5, wheezing, crackle	Asthma, COPD, bronchitis, healthy

*COPD* chronic obstructive pulmonary disease

Recently, many new datasets have emerged for lung sound analysis. Fraiwan et al. [85] collected 112 lung sound recordings from 112 subjects who were healthy or diagnosed with asthma, pneumonia, COPD, bronchitis, heart failure, lung fibrosis, and pleural effusion. Each recording was annotated according to the different lung sound events, including normal, inspiratory, expiratory, crepitations, crackles, and wheezes. Hsu et al. [86] proposed a new dataset called HF_Lung_V1, which consists of 9765 lung sound recordings with a duration of 15 s from 261 subjects. These recordings were collected using a single-channel device (3 M LITTMAN 3200) and a multi-channel device (self-customized device, HF-Type-1). HF_Lung_V1 marked 34,095 inspiratory segments, 18,349 expiratory segments, 13,883 continuous adventitious sound segments, and 15,606 discontinuous adventitious sound segments. Moreover, Hsu et al. [87] collected lung sounds from 42 new subjects to expand HF_Lung_V1 into a new dataset, namely HF_Lung_V2. More details about these public datasets are given in Table 2**.**

In addition, the need for the management of chronic pulmonary disease like COPD has also gradually attracted the attention of clinicians and researchers [89], where the assessment of disease severity is a prerequisite for determining medical interventions [90]. Altan et al. [88] released a dataset called RespiratoryDatabase@TR that collected lung sounds from patients diagnosed with asthma, bronchitis, and different severities of COPD (0–5). In the trial, each subject underwent the examinations of chest X-rays, PFTs, and cardiopulmonary auscultation. The resulting dataset consists of 77 recordings from 77 subjects, with each recording sampled at 4000 Hz and containing 4 channels of heart sounds and 12 channels of lung sounds. For annotation, two pulmonologists validated and labeled the sound records as murmur, crackle, or wheezing, with reference to the gold standards of chest X-rays and PFTs. RespiratoryDatabase@TR has been widely used to assess the severity of COPD [27, 91, 92].

### Data acquisition and preprocessing

**Fig. 5:**
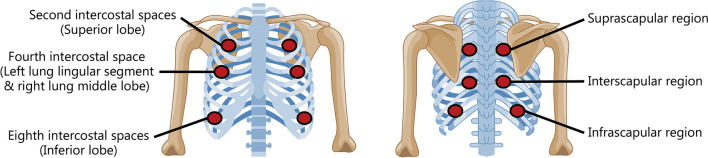
Auscultation sites. The red dots indicate auscultation. Typically, doctors monitor the lungs in a symmetrical way, up and down

In the clinical procedure for acquiring lung sound data, the digital stethoscope should be placed on specific parts of the thoracic surface for certain durations (e.g., 15 s, 30 s, or even longer) to depict the overall lung condition. As shown in Fig. 5, the monitoring of the superior lung lobe requires the digital stethoscope to be placed on both the left and right second intercostal spaces on the anterior chest, along with the suprascapular region at the equivalent horizontal level. The fourth intercostal space and the interscapular region are correspondingly affiliated with the superior lobe of the left lung (the lingular segment) and the middle lobe of the right lung. To assess the inferior lobes of the lung, auscultation should be performed on the left and right eighth intercostal spaces as well as the infrascapular region. Through this process, the lung sound data from the audio recorded by the stethoscope are extracted in the form of electrical signals. However, since lung sound is fragile to environmental noise and the disturbance caused by internal heartbeat sounds, it is necessary to preprocess the raw recordings to ensure that lung sound is the dominant component of the recordings [93]. According to the different noise sources, the preprocessing can be subdivided into two types, namely external noise reduction and heart sound separation.

External noise reduction methods are generally based on three different technologies. (1) Filter-based: this technology has the ability to quickly process a large amount of data but it is difficult to remove noise, with frequency information overlapping with lung sounds [94–96]; (2) Wavelet-based: this can decompose the mixed signal based on its time–frequency information to obtain the denoised signal; however, its denoising effect is easily affected by the selection in the wavelet basis function and threshold function [97–99]; (3) Empirical mode decomposition (EMD) based: this eliminates different types of noise in the audio signal but requires high computational complexity and reasonable parameter selection [100, 101]. For example, Meng et al. [102] decomposed the noisy signal into seven sub-signals using wavelet decomposition and located the position of the lung sound in each sub-signal using autocorrelation coefficients to extract the effective lung sound components. Haider et al. [103] used EMD to decompose the noisy signal and integrated Hurst analysis for intrinsic mode function (IMF) selection to reduce the noise from the lung sound recording. Based on prior knowledge of lung sound signals, Emmanouilidou et al. [11] processed the noisy signal in short-time windows and used the current frame’s signal-to-noise information to dynamically extract the interested components of lung sound.

To separate the lung sound and heart sound, various methods have been proposed based on blind source separation (BSS), such as filter-based methods, independent component analysis (ICA), wavelet-based methods, and non-negative matrix factorization (NMF) [104–109]. Grooby et al. [110] presented an NMF-based method that separates the raw sound recording into both the heart sound and lung sound. Although these methods have shown their effectiveness, the results of ICA-based separation are varied due to the selection of the number of iterations and convergence criteria, resulting in uncertainties in the phase, amplitude, or ranking order of separated signals. In the NMF-based method, the spectrogram of mixed signals is decomposed into two non-negative matrices, minimizing the difference between the product of the two non-negative matrices and the original matrix. Since the minimization process involves non-convex optimization, the decomposed signal is easily limited to the local optimal solution, resulting in poor noise reduction. In addition, the periodicity of heart sound has been applied to differentiate heart sound from lung sound [111, 112]. For example, Ghaderi et al. [113] applied singular spectrum analysis to locate and separate different trends of heart sound and lung sound.

### Feature extraction

**Fig. 6:**
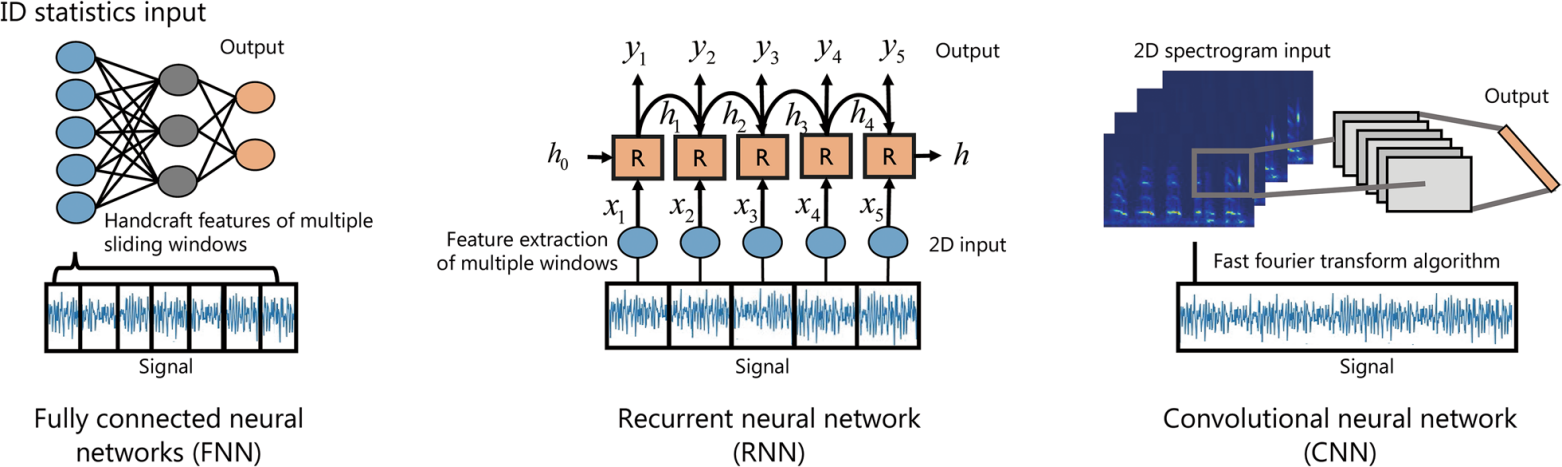
Design procedure of deep learning models. FNN makes predictions based on 1-D statistical features extracted from multiple windows, and RNN predict the health states based on the 2-D features of each window. CNN learns the deep features from the 2D spectrogram input to predict the health states. 1D one-dimensional, 2D two-dimensional

The high variability of lung sound is caused by many factors, such as age, sex, lung disease, and body position. The feature extraction method is important for obtaining distinctive feature representations for classification. As shown in Fig. 6, the representations of lung sound rely on two different types of feature extraction: traditional handcrafted feature extraction and deep learning-based feature extraction [114], which are discussed below.

The traditional handcrafted features have quantifiable characteristics of audio signals that can be used to differentiate various sounds, which can be subdivided as follows: (1) time-domain features, which capture information related to lung sound variations over time, such as zero-crossing rate, root mean square, and signal envelope; (2) frequency-domain features, which provide information about the distribution of energy across various frequency bands, such as spectral centroid, spectral roll-off, and spectral flux. Mel-frequency cepstral coefficients (MFCCs) are a commonly used feature in lung sound analysis derived from the Fourier transform, which can capture the distribution of energy in different frequency bands [115, 116]; and (3) time–frequency domain features, which record the distribution of energy across different frequency bands over time, providing valuable insights into the non-stationary and transient nature of lung sounds, such as wavelet transform and spectrogram [117–119]. Researchers generally use a combination of multiple-domain handcrafted features as representations for lung sound analysis [120]. Among them, the statistical feature is a commonly used combination representation derived from a short temporal sliding window that divides the signal into multiple segments to extract multi-domain features. The statistical values of each feature across multiple segments, such as mean, variance, skewness, and kurtosis, are calculated as the representation. Deep learning-based feature extraction is a data-driven approach that learns features directly from the raw data without the need to design manual features [121–123]. The CNN, with the input of the spectrogram, is commonly used to capture complex and hierarchical patterns within data and can learn more discriminative and robust representations. Pham et al. [124] explored the effect of different types of spectrograms and the spectral-time resolution in deep learning-based lung disease detection. Long short-term memory (LSTM) is another important method for feature extraction based on raw data or frequency-domain features. Fraiwan et al. [125] used CNN to extract the time–frequency information of multiple windows from the raw signal, then used LSTM to mine the continuous time–frequency change information for pulmonary disease recognition.

In summary, traditional handcrafted features are manually designed based on the human understanding of audio signals that emphasize different characteristics of lung sounds in different targeting domains. These handcrafted features are usually easy to interpret and computationally efficient. Initially, the 1D handcrafted features combined with fully connected neural networks (FNNs) were often used for lung sound analysis by projecting the feature vectors into the specified task space [117]. However, handcrafted features are more sensitive to noise, suffering from quality drops when unexpected events emerge (e.g., talking, footsteps, and coughing) [93]. Unlike handcrafted features, deep learning-based feature extraction does not fully rely on the human understanding of acoustics or audio content, but automatically learns the task-relevant features from a large amount of lung sound data. Here, CNN combined with the input of 2D spectrogram representation is the most commonly used method, wherein the spectrogram records the raw signal information in the time–frequency domain, and the convolutional kernel is used to integrate the frequency and time domain features to generate high-level semantic representations. The features learned by the deep learning model have the clear advantage of high complexity and dimensionality; however, they lack interpretability since the procedure of network optimization (e.g., backpropagation) is not transparent. Furthermore, this approach requires more computing resources.

### Deep learning methods

This section outlines the existing deep-learning methods for lung sound analysis [10, 22–27, 33, 72, 77, 82, 91, 92, 117, 122–157], as shown in Table 3. Many aspects of deep learning-based lung analysis are overviewed: basic model selection, the advancement of medical tasks, and limitations and future directions.

**Table 3:** Deep learning methods in lung sound analysis

Task	Year	Ref	Basic method	Dataset	Outcome
ASD	2013	[126]	Wavelets, FNN	Self-collected, 13 healthy, 13 pathological	Normal or crackle: ACC—71.55%
	2014	[117]	Wavelets, FNN	Lehrer's dataset	Normal, wheeze or crackle: ACC—99.26%
	2016	[132]	MFCCs, LFCCs, FNN	RALE database, IIT Kharagpur, Salt Lake, Kolkata	Normal, wheeze or crackle: ACC—97.61%, SEN—97.41%, SPE—98.33%
	2018	[33]	Spectrogram, CNN	RALE dataset	Coarse crackle, fine crackle, polyphonic wheeze, monophonic wheeze, normal, squawk, stridor: ACC—95.56%
	2018	[127]	MFCCs, RNN	Self-collected, 10 healthy, 5 idiopathic pulmonary fibrosis	Inspiration: F1—87%; Expiration: F1—84.6%; Crackles: F1—72.1%
	2018	[147]	LSTM	ICBHI 2017	Normal, crackles, wheezes, and both crackles and wheezes: SEN—58.4%, SPE—73.0%, AS—65.7%
	2019	[133]	Spectrogram, CNN	Self-collected, 50 pediatric patients	Wheeze, rhonchi, fine crackle, or coarse crackles: recall—76.5%, precision—53.0%, SPE—83.6%, F1—62.5%
	2019	[148]	Spectrogram, CNN	CBHI 2017	Normal, crackles, wheezes, and both crackles and wheezes: SEN—31.12%, SPE—68.20%, AS—50.16%
	2020	[23]	Mel spectrogram, CNN	ICBHI 2017	Normal, crackles, wheezes, and both crackles and wheezes: SEN—48.63%, SPE—84.14%, AS—66.38%
	2020	[131]	Mel spectrogram, CNN	ICBHI 2017	Normal, crackles, wheezes, and both crackles and wheezes: AS—78.4%; normal and abnormal: AS—83.7%
	2020	[136]	Mel Spectrogram, CNN	ICBHI 2017	Normal, wheeze or crackle: ACC—98.6%, F1—98.4%
	2020	[149]	Spectrogram, LSTM, CNN, autoencoder	Self-collected, 22 patients	Inspiration or expiration: ACC—92%
	2020	[150]	Spectrogram, CNN	Self-collected, 25 pediatric patients	Normal, wheeze or crackle: crackle PPA—95%, NPA—99%; wheeze PPA—90%, NPA—97%
	2020	[151]	Spectrogram, CNN	RALE database, Think labs Lung sound library	Normal, crackles, wheezes, or rhonchi: ACC—83.78%
	2021	[22]	Spectrogram, CNN	ICBHI 2017	Normal, crackles, wheezes, and both crackles and wheezes: SPE—85.44%, SEN—70.93%, AS—78.18%
	2021	[72]	Mel spectrogram, CNN	ICBHI 2017	Normal, crackles, wheezes, and both crackles and wheezes: SPE—72.3%, SEN—40.1%, AS—56.2%; Normal or abnormal: SPE—80.9%, SEN—73.1%, AS—77.0%
	2021	[77]	CNN	Self-collected, 1918 respiratory sound record	Normal, abnormal (crackles, wheezes, rhonchi): ACC—84.8%, precision—81.4%, recall—81.7%, F1—81.4%
	2021	[152]	Spectrogram, CNN, autoencoder	ICBHI 2017	Normal, crackles, wheezes, and both crackles and wheezes: SPE—69%, SEN—29%, AS—49%
	2023	[82]	Spectrogram, CNN	Self-collected, 105 health, 189 patients	Normal, crackles, or rhonchi: ACC—83%
	2022	[130]	Mel spectrogram, CNN	ICBHI 2017	Normal, crackles, wheezes, and both crackles and wheezes: ACC—84.7%, SEN—84.5%, SPE—84.9%, precision—84.4%, recall—89.0%, F1—86.6%
	2022	[134]	Spectrogram, CNN	ICBHI 2017	Normal, crackles, wheezes, and both crackles and wheezes: SPE—85.6%, SEN—29%, AS—57.3%
	2022	[135]	Mel spectrogram, TCN	ICBHI 2017	Normal, crackles, wheezes, and both crackles and wheezes: SPE—86.1%, SEN—65.3%, AS—75.7%
	2022	[137]	LSTM, CNN	ICBHI 2017	Normal, crackles, wheezes, and both crackles and wheezes: SPE—82.46%, SEN—47.37%, AS—64.92%
	2022	[146]	Spectrogram, Mel spectrogram, CNN	ICBHI 2017	Crackles or others: ACC—86.4%; Wheezes or others: ACC—78.2%; Crackles, wheezes, or others: ACC—84.5%
RDR	2013	[153]	Statistical feature, FNN	Self-collected, 27 healthy and 33 tuberculosis	Healthy or pulmonary tuberculosis subject: ACC—73%
	2014	[138]	FNN	Self-collected, 10 healthy and 20 pathological	Normal and abnormal subject: ACC—92.86%, SEN—86.30%, SPE—86.90%
	2018	[27]	Deep belief networks	RespiratoryDatabase@TR	Risk level or interior level: ACC—95.84%, SEN—93.34%, SPE—93.65%
	2018	[91]	Extreme learning machines	RespiratoryDatabase@TR	COPD or health: ACC—92.22%, SEN—89.44%, SPE—95.00%
	2018	[154]	Deep extreme learning	RespiratoryDatabase@TR	COPD or health: ACC—95.0%, SEN—93.33%, SPE—93.53%
	2019	[123]	Spectrogram, CNN	ICBHI 2017	All diseases classification: ACC—97%
	2020	[155]	Boltzmann machines	RespiratoryDatabase@TR	COPD or healthy subject: ACC -93.67%, SEN—91%, SPE—96.33%
	2020	[122]	Convolutional RNN	Self-collected, 16 healthy and 7 pulmonary fibrosis	Health or idiopathic pulmonary fibrosis: precision—100%, SEN—85.9%, F1—92.4%
	2020	[139]	Mel spectrogram, CNN	ICBHI 2017	Non-COPD, COPD, or healthy subject: SEN—98.5%, SPE—99.0%, AS—98.7%
	2020	[143]	Extreme learning machines	RespiratoryDatabase@TR	Five severity degrees of COPD: ACC—94.31%, SEN—94.28%, SPE—98.76%
	2020	[156]	Statistical feature, FNN	ICBHI 2017	Health or diseases: ACC—82%, precision—87%
	2021	[140]	EMD, wavelet, CNN	ICBHI 2017	Non-COPD, COPD, or healthy subject: precision—98.90%, recall—98.90%, ACC—98.92%, F1—98.90%; Six diseases: precision—98.70%, recall—98.27%, ACC—98.70%, F1—98.47%
	2021	[144]	Deep belief network	RespiratoryDatabase@TR	Mild, moderate, or severe COPD: ACC—71.74%, SEN—70.08%, SPE—73.53%
	2022	[125]	CNN, LSTM	Self-collected, 103 patients	Health or five diseases: ACC—98.16%, SEN—90.06%, SPE—98.61%, precision—92.13%
	2022	[141]	Wavelet, CNN, LSTM	ICBHI 2017	Health, COPD, asthma, or pneumonia: ACC—88.86%; Health, COPD, or non-COPD: ACC—66.54%
	2022	[142]	FNN, CNN, LSTM	ICBHI 2017	URTI, COPD, pneumonia, and bronchiolitis and healthy: ACC—94%, precision—94%, recall—94%, F1—93%
	2022	[157]	Statistical feature, FNN	ICBHI 2017	Health or diseases: SPE—97.6%, SEN—98.2%
	2023	[10]	CNN, LSTM	Self-collected, 198 patients	Disease, symptom relief, or health: 1) subject-dependent: SEN—96.98%, SPE—97.43%, AS—97.20% 2) subject-independent: SEN—43.26%, SPE—39.61%, AS—41.44%
	2023	[92]	Mel spectrogram, pretrained MobileNet-V1	RespiratoryDatabase@TR	Risk level or interior level: ACC—99.25%, SEN—99.18%, SPE—99.36%; Five severity degrees of COPD: ACC—96.14%, SEN—95.94%, SPE—98.89%
	2023	[129]	CNN	Self-collected, 126 subject	Health, asthma, COPD, ILD, pneumonia, bronchiectasis: precision—92.81%, SEN—92.22%, SPE—98.50%
ASD, RDR	2019	[26]	MFCCs, LSTM	ICBHI 2017	Normal, crackles, wheezes, and both crackles and wheezes: ACC—74%, SPE—85%, SEN—62%, AS—74%; Normal or abnormal: ACC—81%; Health or diseases: ACC—99%, SPE—82%, SEN—99%, AS—91%; Health, COPD, or non-COPD: ACC—98%, SPE—82%, SEN—98%, AS—90%
	2020	[25]	Spectrogram, CNN	ICBHI 2017	Normal, crackles, wheezes, and both crackles and wheezes: SPE—89%, SEN—72%, AS—80%; Normal or abnormal: SPE—89%, SEN—82%, AS—85%; Health or diseases: SPE—71%, SEN—99%, AS—85%; Health, COPD, or non-COPD: SPE—71%, SEN—95%, AS—83%
	2021	[124]	Spectrogram, CNN	ICBHI 2017	Normal, crackles, wheezes, and both crackles and wheezes: SPE—90%, SEN—68%, AS—79%; Normal or abnormal: SPE—90%, SEN—78%, AS—84%; Health or diseases: SPE—86%, SEN—98%, AS—92%; Health, COPD, or non-COPD: SPE—86%, SEN—95%, AS—91%
	2021	[128]	Mel spectrograms, CNN	ICBHI 2017	Normal, crackles, or wheezes: SPE—82%, SEN—61%, AS—72%; COPD, healthy, and pneumonia: SPE—92%, SEN—98%, AS—95%
	2021	[145]	Spectrogram, Inception	ICBHI 2017	Normal, crackles, wheezes, and both crackles and wheezes: SPE—73%, SEN—30%, AS—52%; Health, COPD, or non-COPD: SPE—100%, SEN—75%, AS—85%
	2022	[24]	Spectrogram, CNN	ICBHI 2017	Normal, crackles, wheezes, and both crackles and wheezes: SPE—78.55%, SEN—35.97%, AS—35.97%; Normal or abnormal: SPE—79.34%, SEN—50.14%, AS—64.74%; Health, COPD, or non-COPD: SPE—91.77%, SEN—93.68%, AS—92.72%; Health or diseases: SPE—91.77%, SEN—95.76%, AS—93.77%

*ACC* accuracy, *AS* average score of specificity and sensitivity, *ASD* abnormal sound detection, *COPD* chronic obstructive pulmonary disease, *CNN* convolutional neural network, *EMD* empirical mode decomposition, *FNN* fully connected neural network, *ILD* ınterstitial lung disease, *LFCCs* linear frequency cepstral coefficients, *LSTM* long short-term memory, *MFCCs* mel-frequency cepstral coefficients, *NPA* negative percent agreement, *PPA* positive percent agreement, *RDR* respiratory disease recognition, *RNN* recurrent neural network, *SEN* sensitivity, *SPE* specificity, *IIT* Indian Institute of Technology*, F1* F1-score*, URTI* upper respiratory tract infection

#### Basic model selection

The construction of a specific deep-learning model is based on the structure of input data, as shown in Fig. 6. FNNs can be used to extract information from a 1D representation, such as the 1D statistical features of lung sound data. For RNNs, the lung sounds will be divided into continuous time windows, and the acoustic features will be extracted from each window to form a 2D lung sound representation. Then, the RNN uses the hidden layer to learn the temporal changes of lung sounds for disease classification. CNNs are more suitable for 2D data representation, such as images (e.g., 2D spectrograms of lung sound). Therefore, the construction of deep learning can be done based on the selection of a specific deep learning model according to its input structure. The basic models can be referred to [33, 126, 127]. Preferably, the model undergoes some tailoring or tuning of its structure based on the classification task and optimization strategy [24, 128, 129]. For example, the FNN-based method transforms the lung sound into a combination representation of acoustic characteristics, then feeds it to the FNN for abnormal sound identification [18]. Charleston-Villalobos et al. [118] extracted power spectral density as the representation of lung sound, then used a FNN to distinguish between healthy subjects and interstitial lung disease (ILD) patients, achieving a mean accuracy of 84% with a self-collected dataset. The RNN-based method analyzes the temporal dynamics of lung sounds, which provides insight into the progression of respiratory diseases over time [127]. Perna et al. [26] exploited the temporal information of lung sounds by using an RNN to recognize abnormal lung sounds, achieving 85% specificity and 62% sensitivity. The CNN-based method learns the temporal-frequency features from the 2D spectrogram of lung sounds to detect abnormal patterns and infer health conditions [33, 121]. Based on the ICBHI 2017 dataset, Yu et al. [130] extracted global and local features from the Mel spectrogram with a CNN to recognize normal lung sounds, crackle, wheeze, and both, achieving 84.9% specificity and 84.5% sensitivity.

#### Advancement of medical tasks using lung sound analysis

For medical purposes, deep learning methods can be sorted for two main tasks. (1) ASD: this is a diagnostic auxiliary task that involves the detection of specific abnormal lung sounds, usually crackling and wheezing, as the basis for the diagnosis of specific diseases; and (2) respiratory disease recognition (RDR): this is an automated diagnostic task that directly distinguishes respiratory patients from healthy subjects or identifies patients with different types of respiratory diseases, such as patients with COPD, pneumonia, and asthma. The relationship between them is shown in Fig. 4**.**

ASD consists of two sub-tasks:

1. 2-classes abnormal lung sound detection. As a binary classification, this focuses on distinguishing abnormal lung sounds from normal lung sounds without concrete labels or on detecting one type of abnormal lung sound (e.g., crackle, wheeze, and stridor). Serbes et al. [126] explored the effect of different wavelet types and window sizes in FNN-based crackle detection, where Gaussian, Hanning, Hamming, and Rectangular windows were considered, while Morlet, Mexican Hat, and Paul wavelets were applied to lung sound recognition. Nguyen et al. [131] proposed the methods of temporal stretching and vocal tract length perturbation for data augmentation to solve the issue of limited training samples, then used a CNN as the backbone for abnormal lung sound detection.
2. Multi-classes abnormal lung sound recognition. This is used to distinguish between specific abnormal sounds including crackles, wheezes, and rhonchi, where the number of classes is dependent on the number of types of abnormal sounds. Sengupta et al. [132] extracted statistical features based on MFCCs for lung sound, then fed a FNN to distinguish normal, wheeze, and crackle sounds. Their experiment was carried out on 30 subjects and showed that MFCC-based statistical features outperformed wavelet-based features in finding abnormal sounds. Bardou et al. [33] extended the types of abnormal lung sounds to include normal, coarse crackle, fine crackle, monophonic wheeze, polyphonic wheeze, squawk, and stridor, then used a spectrogram-based CNN to identify these types. Grzywalski et al. [133] conducted a clinical trial to compare the accuracy of abnormal lung sound detection between an artificial intelligence (AI) algorithm and doctors, where a CNN was trained to detect four types of lung sound: wheezes, rhonchi, and fine and coarse crackles. This trial suggested that CNN-based abnormal lung sound detection is more accurate than doctors in regard to the metrics of sensitivity and F1-score. With the release of the ICBHI 2017 dataset, the number of studies on ASD for detecting normal sound, crackles, wheezes, and both crackles and wheezes exploded [23, 130, 134, 135]. Rocha et al. [136] separately trained a classifier for crackle detection, wheeze detection, and mixture detection (crackle, wheeze, and others) and used four different machine learning methods to evaluate its effectiveness (e.g., boosted trees, SVM, and CNN). Gairola et al. [72] proposed a concatenation-based augmentation to solve the unbalanced class issue, and used the ResNet block for abnormal lung sound detection. For a limited training sample, Song et al. [22] proposed an abnormal lung sound detection method that encourages intra-class compactness and inter-class separability by comparing samples from different classes during the training phase. To explore the temporal and frequency information of lung sound, Petmezas et al. [137] integrated a CNN and an RNN for abnormal lung sound detection, where the former extracts the deep temporal-frequency features from spectrograms, and the latter uses the deep features to mine the change of lung sound over the time.

For RDR, most studies were evaluated on ICBHI 2017 and focused on four sub-tasks:

1. 2-classes respiratory pathology recognition. This is used to distinguish patients from healthy people. Messner et al. [122] collected lung sounds from healthy subjects and patients with idiopathic pulmonary fibrosis, then applied a convolutional RNN to lung sound analysis for binary classification (e.g., healthy vs. pathological). Mondal et al. [138] extracted the statistical feature combination of kurtosis, sample entropy, and skewness from lung sounds and used FNN to infer lung health conditions.
2. 3-classes respiratory chronic disease recognition. This divides populations into three groups: healthy subjects, chronic patients (e.g., COPD, bronchiectasis, and asthma patients), and non-chronic patients (e.g., those with upper and lower respiratory tract infection, pneumonia, and bronchiolitis). García-Ordás et al. [139] converted lung sounds into Mel spectrogram representations to train CNNs to recognize respiratory pathologies, meanwhile using variational autoencoders to generate new samples for minority classes to solve the issues of unbalanced data. Shuvo et al. [140] decomposed the preprocessed signal using EMD to obtain an IMF signal that had a high correlation with the lung sound signal, then applied the continuous wavelet transform to extract a discriminative representation for training a lightweight CNN model. Their proposed method was evaluated on ICBHI 2017 and outperformed other lightweight models. Shi et al. [141] explored the temporal-frequency information of different scales with the dual wavelet analysis module, and used the attention module to extract the salient difference information for respiratory chronic disease recognition.
3. Multi-types specific RDR. This task is used to distinguish between specific respiratory diseases (e.g., COPD, asthma, and pneumonia), where the number of classes depends on the total class of the disease. Tariq et al. [123] applied a variety of data augmentation methods to solve the issue of unbalanced classes (e.g., time stretching, pitch shifting, and dynamic range compression) and used a CNN to extract pathological features from the spectrogram to recognize seven respiratory diseases. Kwon et al. [142] explored the performance of different combinations of feature extraction methods and classifiers in detecting lung conditions (e.g., healthy lungs, Upper respiratory tract infection, COPD, pneumonia, and bronchiolitis).
4. Multi-courses respiratory disease severity recognition. This task aims to distinguish the severity of respiratory diseases, in which the number of classes generally depends on the medical definition of disease progression. Morillo et al. [158] adopted principal component analysis and FNN to detect whether COPD patients were aggravated by pneumonia, with a sensitivity and specificity of 72.0% and 81.8%, respectively. Based on the RespiratoryDatabase@TR dataset, Altan et al. [27] proposed the method of using a 3D-second order difference plot to analyze lung sound signals, then using pre-trained deep belief networks to distinguish the risk level from the interior level for COPD patients. This approach demonstrated the validity of pre-trained deep-learning architectures in RDR. Huang et al. [10] proposed a hybrid model based on pre-trained VGGish networks and BiLSTM to identify the severity of community-acquired pneumonia among children, including pneumonia-confirmation, spontaneous resolution, and recovery. Altan et al. [143] adopted the cuboid and octant-based quantization methods to extract characteristic abnormalities from a 3D-second order difference plot, then used a deep extreme learning machine classifier to separate five COPD severities. Yu et al. [144] explored the ability of multiple methods (SVM, decision tree, and deep belief network) to identify the severity of COPD, where the deep belief network achieved 93.67% accuracy in distinguishing between patients with mild, moderate, and severe COPD.

More recently, some studies proposed deep learning-based methods that can be used for both RDR and ASD [25, 124, 145], as shown in Table 3. Perna et al. [26] extracted the MFCCs of multi-window from lung sound signals to generate representations, then used an RNN-based model. Li et al. [128] proposed a knowledge distillation-based method that transfers the weights of a CNN learned from multiple centers into a fuzzy decision tree, which provides an interpretable model for abnormal lung sound detection and chronic RDR. Nguyen et al. [24] introduced different methods to adapt a pre-trained model to a new environment, including fine-tuning, co-tuning, stochastic normalization, and their combination, for ASD and RDR. In their experiments, the authors noted that varying performance was caused by differences in equipment and introduced spectrum correction to solve this issue [159].

### Limitations and future directions

Table 3 summarizes the state-of-the-art deep learning approaches for ASD and RDR. It shows that most methods use specificity, sensitivity, and the confounding index between the two for ASD, while evaluation metrics (e.g., accuracy, precision, recall, and F1) are added based on the evaluation metrics of ASD for RDR. In terms of the model, a CNN with the input of a spectrogram and Mel spectrogram is currently the most widely-used method for both tasks, achieving over 80% specificity and 60% sensitivity in the ICBHI 2017 dataset for ASD and having over 90% accuracy, recall, precision, and F1 for RDR. In addition, most methods recently used a structure that applies a CNN to extract deep features from multiple consecutive temporal windows, then uses the deep features of successive windows as the input of RNN to learn the contextual information for RDR. Table 3 shows that deep learning has made progress regarding lung sound-based medical tasks, demonstrating the capability to identify different abnormal sounds, pulmonary diseases, and disease severity. However, the clinical application of deep learning-based lung sound analysis still faces some challenges, as discussed below.

The main challenge is that most deep learning-based lung sound analysis methods have poor interpretability [128]; thus deep learning-based methods currently only play a supporting role in clinical applications. Specifically, physicians rely on the interpretation of lung sounds for medical decision-making. However, the black-box operation of deep learning makes it difficult for physicians to understand how the model works in the diagnosis, that is its mechanism is not fully clear. As a result, physicians cannot fully trust or rely on the results given by the model. Potential solutions to improve interpretability include the following. (1) Symptom localization: intuitively, the segmentation network can highlight the segments of lung sound in the respiratory cycle to locate the symptoms caused by the disease. These segments can be used not only for disease diagnosis, but also for physicians to confirm the final outcome based on intermediate supporting results [160]. The appearance and localization of abnormal sounds in specific respiratory diseases can be exploited as the trigger of intelligibility by combining them with clinical knowledge; (2) Input visualization: Gradient-weighted class activation mapping analyzes input and gradients to generate interpretable heatmaps that can be used to understand which regions the model focuses on when making decisions [161]. This can present the intermediate results of the model during the decision-making process, which may convince the clinician of its reliability [162]; (3) Knowledge distillation: this can distill the knowledge learned from complex models to another model with interpretability, such as decision trees or linear regression, to achieve an interpretable recognition process with high performance [128]; (4) Surrogate model: this generates a simple, interpretable local model for each specific input to approximate the behavior of the original complex model given the input, such as local interpretable model-agnostic explanations (LIME) [163]. Thus, LIME can help explain the predictions of complex models on specific inputs.

Another challenge is that deep learning-based lung sound analysis lacks robustness under some conditions. (1) Noise sensitivity: most methods have performance degradation due to an increased noise level [136], meaning that the reliability of deep learning methods will be compromised in disease diagnosis due to distortions, resulting in misdiagnosis and missed diagnosis; (2) Device difference: due to the difference between devices regarding sensors, timbre, and sound quality, the performance of a model trained on a single device will fluctuate or drop when tested on other devices [23, 24]; (3) Physiological diversification: Fernandes et al. [146] reported that physiological differences between patients, including age, sex, and body mass index, caused deviations in the performance of models for ASD. To address this problem, transfer learning which mines invariant features under different factors (e.g., noise, devices, and physiological differences) for lung sound analysis, may be an option. It can map the data with differences into aligned data distributions to improve generalizability [164, 165]. Moreover, multi-input models that take these differences as input and force the model to dynamically adjust its weight based on the input to improve generalizability may be effective.

In addition, due to differences in the morbidity of pulmonary diseases, the data distribution of lung sound is a long-tail distribution, which may cause the poor recognition ability of models for rare categories. Most methods adopt data augmentations to address this issue [22, 72, 139]; however, they are still unreliable in real clinical applications since the data augmented by perturbations are different from patient data in practice. To address this issue, few-shot learning might be a useful tool that aims to extract the representative features from a limited number of training samples to exhibit good generalization when faced with new, unseen data [166]. For example, prototypical networks achieved remarkable results in audio event classification with the long-tail distribution [167, 168]. The key idea is to learn the prototype representation of each class, then perform the classification by calculating the distance between the new sample and each prototype [169]. In addition, contrastive learning can be applied to lessen long-tail distribution issues by increasing the distance between different classes in the feature space. Li et al. [170] integrated the idea of prototypical networks to first generate a set of targets uniformly distributed on a feature space, then make the features of different classes converge to these distinct and uniformly distributed targets during training. This forces all classes, including a few, to remain uniformly distributed by the constraints of targeted supervised contrastive learning on the feature space during the optimization process to improve class boundaries.

It is worth noting that most existing lung sound studies only focus on accuracy rather than taking computational resource consumption into account, tending to use models with a large number of parameters that demand more memory and high computational resources [6, 14, 122]. This poses challenges to implementation on the chips of portable devices with limited computation power as compared to servers or personal computers, especially considering the cost-effective hardware solutions that are important for large-scale deployment in poor-resource areas for healthcare improvement. The edge computing of intelligent stethoscopes allows the processing of lung sound data on the device, which reduces the time delay in decision-making and monitoring caused by data transmission in cloud computing, protects the privacy of patients, and reduces the cost of maintaining the cloud server. Such a device is also suitable for disease or well-being management at home by tracking and predicting recovery. Therefore, we consider portable digital stethoscopes equipped with deep learning methods to be a major research direction in this field. Here, we present three strategies to embed deep learning models into the chip of a stethoscope for edge computing. (1) Lightweight model: a large number of methods, such as knowledge distillation and pruning, have been used to lightweight large-scale models to reduce computational requirements [171]; (2) Hardware acceleration: characteristics of hardware, such as parallel processing capabilities, high-speed memory access, and customized computation units, are proven to accelerate computation in deep models [172]; and (3) Operational optimization: the complexity and computation of deep models can be dropped by optimizing basic operators (e.g., depthwise separable convolution decomposes the convolution operation into two separate layers, a depthwise convolution layer and a pointwise convolution layer) [173]. With the above three strategies, deep learning models can be implemented in the chips of digital stethoscopes in the near future, turning the devices into intelligent stethoscopes that not only make recordings of lung sounds, but also give prompt predictions on potential diseases, which can better assist clinicians in consultation.

## Open-source framework

Due to the poor reproducibility caused by the variety of deep learning methods, an open-source framework intended to build a solid foundation for replication and extension has been released to facilitate progress in this field. This framework provides the commonly used methods (e.g., FNN with acoustic feature input and CNN with spectrogram input) and demonstrates them on the ICBHI 2017 dataset as an example of benchmarking. In addition, the framework decomposes the algorithm into four major modules: preprocessing for segmentation and noise reduction, feature extraction for input representation, evaluation metrics for performance assessment, and classifier design for training and testing. Thus, researchers can focus on improving specific steps while keeping the rest identical, which can largely improve the efficiency and agreement of the benchmark. This framework was developed based on PyTorch, and each module contains a main function that is called upon to execute the corresponding task.

The preprocessing module consists of two main operations: (1) Noise suppression. Since lung sounds are easily contaminated in the real environment, this framework executes basic noise suppression based on the band-pass filter to retain the frequency band information of interest for lung sounds. In addition, it provides candidates for noise suppression, including EMD, wavelet denoising, ICA, etc. (2) Segmentation. This step segments the input audio recording into intervals to form a uniform input to train the deep model. For the ICBHI 2017 dataset, each audio recording has each respiratory cycle annotated, i.e., the cycles with abnormal lung sounds (crackles and wheezes) are annotated as 1 and the other as 0. This module splits the recording with such labels. If the duration of the segment is insufficient, smart padding [131] or zero padding is used.

The feature extraction module transforms the 1D sound signal into a representation suitable for the model input. For FNNs and RNNs, lung sound analysis methods adopt the statistical features extracted from segmentation as the representation to train and test the model. This framework performs extraction using pyAudioAnalysis [174]. For CNNs, spectrogram-based input is generally employed for training and testing, where the framework uses the Librosa library to extract different spectrograms, including the Mel spectrogram.

The evaluation metrics module provides the data-splitting strategies and the commonly used evaluation metrics for the experiment setting. To date, there are two data-splitting strategies for lung sound analysis: (1) subject-dependent experiment [22, 130, 131] that randomly splits the entire dataset into training and testing sets. Here, the data from one subject exist in both the training set and the testing set; and (2) subject-independent experiment [10, 24, 175] that splits the entire dataset into training and testing sets in a subject-wise manner. Here, the data from one subject only appear in the training set or testing set to implement the cross-subject benchmark. The choice of evaluation metrics has been referred to [84], including accuracy, specificity, sensitivity, and ICBHI score.

The classifier design module is based on PyTorch to automate lung sound analysis, where the training and testing set is loaded based on different dataset splitting strategies. This module is formed by the model design, evaluation metrics, training and testing function, and recording function. For model design, a commonly used basic model is implemented (e.g., FNN, CNN, and RNN). For evaluation metrics, specificity, sensitivity, and the ICBHI score (the mean of specificity and sensitivity) are applied to evaluate the performance of the model according to previous studies [84]. The recording function is applied to visualize the training information including loss, specificity, and sensitivity.

To develop and evaluate deep learning methods, the above modules can be used as a basis or starting point, providing general functional performance as demonstrated on the ICBHI 2017 dataset. Customized functions can be added on top of each module in future research.

## Conclusions

This review provides a systemic overview of the development of deep learning-based lung sound analysis for intelligent stethoscopes. Deep learning has shown effective performance in detecting, classifying, and assessing respiratory conditions from lung sound recordings, especially the CNN model with 2D spectrogram-based input. While there are still challenges to be addressed, including noise reduction, the interpretability of the model, and the robustness of performance, the potential benefits of deep learning-based lung sound analysis are significant regarding the intelligent stethoscope. With further development and refinement, we expect deep learning to empower the digital stethoscope for automatic and intelligent diagnosis. In addition, it can be a part of 5G telemedicine based on video and audio streams, where deep learning-based intelligent stethoscopes provide in-body information (e.g., lung sound and heart sound) and the video provides out-body information (e.g., affective and pain level).

## Abbreviations

ACC: Accuracy
AS: Average score of specificity and sensitivity
ASD: Abnormal sound detection
BSS: Blind source separation
COPD: Chronic obstructive pulmonary disease
CNN: Convolutional neural network
EMD: Empirical mode decomposition
FNN: Fully connected neural network
ILD: Interstitial lung disease
ICA: Independent component analysis
LFCCs: Linear frequency cepstral coefficients
LSTM: Long short-term memory
MFCCs: Mel-frequency cepstral coefficients
NMF: Non-negative matrix factorization
NPA: Negative percent agreement
PPA: Positive percent agreement
RDR: Respiratory disease recognition
RNN: Recurrent neural network
SVM: Support vector machine
SEN: Sensitivity
SPE: Specificity
1D: One-dimensional
2D: Two-dimensional
3D: Three-dimensional
